# Effects of Lumbar Plexus Block Combined with Infiltration Anesthesia on Anesthesia Comfort Scores and Stress Responses in Elderly Patients Undergoing Hip Replacement

**DOI:** 10.1155/2022/8692966

**Published:** 2022-08-10

**Authors:** Jizheng Zhang, Yi Li, Xiaohua Sun, Wanlu Ren

**Affiliations:** Department of Anesthesiology, Tianjin Hospital, 300211 Tianjin, China

## Abstract

**Objective:**

To investigate the effects of lumbar plexus block combined with infiltration anesthesia on anesthesia comfort scores and stress responses in elderly patients undergoing hip replacement.

**Methods:**

The materials of 100 elderly patients undergoing hip replacement who were treated in our hospital (January 2020-January 2021) were retrospectively analyzed, and they were equalized into the experimental group (*n* = 50) and control group (*n* = 50) according to the anesthesia methods. The experimental group received lumbar plexus block combined with infiltration anesthesia, and the control group received combined spinal-epidural anesthesia combined with infiltration anesthesia. The patients' anesthesia comfort scores, stress responses, and postoperative pain indexes were compared between the two groups.

**Results:**

Compared with the control group, the experimental group achieved much lower scores of mood change, shivering response, and traction reaction (*P* < 0.001), indicating that the anesthesia comfort in the experimental group was higher. Compared with the control group, the experimental group had much better perioperative stress response indexes (*P* < 0.05) and eminently lower pain scores at 12 and 24 hours after surgery (*P* < 0.05).

**Conclusion:**

Lumbar plexus block combined with infiltration anesthesia can relieve the stress responses and postoperative pain of elderly patients undergoing hip replacement and increase their anesthesia comfort. Therefore, this anesthesia method is translational in clinic.

## 1. Introduction

With a larger aging population in China, the number of elderly patients undergoing hip replacement because of femoral neck fracture or femoral head necrosis is gradually increasing [1]. Although hip replacement can effectively reduce the disability rate, it has such disadvantages as large intraoperative blood loss and strong postoperative pain [2]. Especially for elderly patients who are complicated with cardiovascular, pulmonary, or respiratory diseases, their organ function declines to some extent, and their stress responses to anesthesia and surgery are more remarkable [3, 4]. Some patients resist early functional training due to pain and prolonged lying-bed period, which causes such complications as deep venous thrombosis and pressure ulcers [5]. These complications seriously affect the patients' postoperative recovery. Therefore, it is important to select appropriate anesthetic measures to enhance intraoperative comfort and reduce postoperative pain in elderly patients. According to previous studies, multimodal balanced analgesia has superior effects on elderly patients [6]. Multimodal balanced analgesia is not only the simple combination of different anesthetic drugs but also refers to the combined use of analgesic drugs or analgesic methods with different mechanisms of action. These drugs or methods have complementary effects due to different mechanisms of action, which can play an analgesic or synergistic effect. At the same time, the reduced dose of each drug can effectively alleviate side effects to meet the maximum ratio of effects to side effects and maintain a dynamic balance. This analgesia has been widely used in clinic in recent years. General anesthesia, lumbar plexus block analgesia, combined spinal-epidural anesthesia, local infiltration anesthesia, and patient-controlled analgesia pump are all common multimodal analgesic methods. General anesthesia, as a traditional type of anesthesia, has the advantages of high comfort and mild stress response but is likely to suppress the patients' respiratory function. Combined spinal-epidural anesthesia can compensate for the disadvantages of general anesthesia but applying anticoagulant drugs in elderly patients during the perioperative period increases the risk of epidural analgesia. Lumbar plexus block analgesia only acts on the peripheral area of the nerves and does not affect the sympathetic nerve, so it is conducive to maintaining hemodynamic stability. However, when the range of hip replacement extends the lumbar plexus, it is needed to combine the lumbar plexus block analgesia with other types of anesthesia in practice [7, 8]. Local infiltration anesthesia refers to the stratified injection of local anesthetics along the surgical incision line to block the nerve endings in tissues. This anesthesia, with higher security and effectiveness, can effectively relieve the patients' early postoperative pain, decrease their dosage of opioids, and reduce the occurrence rate of complications [9]. At present, there are studies reporting the effect of the combination of local infiltration anesthesia, general anesthesia, and patient-controlled analgesia pump, but there is still a gap in the study on the application of local infiltration anesthesia combined with lumbar plexus block or combined spinal-epidural anesthesia in hip replacement. It is still unclear in academic circles which type of analgesia is more beneficial to reduce elderly patients' stress responses. Based on this, this paper investigates the effects of lumbar plexus block combined with infiltration anesthesia on elderly patients undergoing hip replacement, aimed at providing a theoretical basis for the clinical application of this anesthesia method.

## 2. Materials and Methods

### 2.1. Research Design

This was a retrospective study conducted in our hospital from January 2020 to January 2021, aimed at investigating the effects of lumbar plexus block combined with infiltration anesthesia on the anesthesia comfort scores and stress responses in elderly patients undergoing hip replacement. The study adopted the double-blind method, so neither the study subjects nor the investigators were aware of the trial grouping, and none of the subjects knew which group they belonged to, and the study designers were responsible for arranging and controlling all the trials.

### 2.2. General Data

This study included 100 patients, and they were equalized into experimental group and control group according to the anesthesia methods, with 50 patients in each group. The experimental group received lumbar plexus block combined with infiltration anesthesia, and the control group received combined spinal-epidural anesthesia combined with infiltration anesthesia.

Inclusion criteria are as follows: (1) Patients had received medical treatment, had no anesthesia contraindications and no allergic history to anesthetic drugs, and met the indications of hip surgery for elderly patients [10]; (2) patients had no history of hip replacement; (3) patients were over 60 years old; (4) patients were treated at our hospital for the whole course and had complete clinical data; (5) patients had no serious complications and all complications could be effectively controlled; and (6) patients were in the American Society of Anesthesiologists (ASA) [11] grades I-III.

Exclusion criteria are as follows: (1) Patients could not communicate with others because of hearing impairment, language handicap, unconsciousness, mental illness, or other factors; (2) patients had severe organic lesions or serious dysfunction of important organs; (3) patients were under 60 years old; (4) patients' clinical data were lack or lost; (5) patients had surgical contraindications; (6) patients had allergic history to anesthetic drugs; (7) patients suffered from the parasecretion of the adrenal cortex; (8) time from the happening of hip joint fracture to receiving hip replacement was more than 14 days; and (9) patients suffered from multiple fractures.

After collecting, analyzing, and comparing the patients' sociodemographic data and clinical data, no remarkable difference in general data between the two groups was found, and the patients in the two groups could be taken as the study objects, as illustrated in Table 1.

### 2.3. Moral Consideration

This study conformed with the principle of the *Declaration of Helsinki (2013)* [12]. The patients were informed of the contents, significance, purposes, and confidentiality of this study and signed the informed consent.

### 2.4. Methods

The experimental group received lumbar plexus block combined with infiltration anesthesia. (1) Lumbar plexus block: the diseased side was given the ultrasound-guided (GE Healthcare, Voluson P6, NMPA (I) 20152062178) lumbar plexus block by using the out-of-plane technology with the longitudinal scan. After positioning, the needle was inserted in the middle of the ultrasound probe. When the needle tip was observed to be beyond the transverse process, the anesthetist should confirm that there was no blood after pumpback. After that, 25 ml of anesthetic drugs, 0.5% ropivacaine (Guangdong Jiabo Pharmaceutical Co., Ltd., NMPA approval no. H20113381) and 1.0% lidocaine (Jumpcan Pharmaceutical Group Co., Ltd., NMPA approval no. H32023273), were injected. At first, 5 ml of anesthetic drugs were injected and the patients were observed for 3 minutes. Then, the remaining drug was injected around the lumbar plexus when the ultrasound showed no indication of arterial and venous blood flow next to the puncture needle and the patient had no adverse reaction. After successfully operating the nerve block, the patients received 0.5 *μ*g/kg of dexmedetomidine (Jiangsu Hengrui Medicine Co., Ltd., NMPA approval no. H20090248) by intravenous infusion for 10 minutes. When the patients fell asleep, they inhaled sevoflurane through a laryngeal mask and continued to receive 0.3-0.5 *μ*g/(kg·h) of dexmedetomidine by intravenous infusion until 0.5 hours before the end of surgery. (2) Infiltration anesthesia: after fitting the prosthesis, the patients were given 2.5 g (80 ml) of ropivacaine (containing 0.1 mg of epinephrine) by infiltration injecting around the articular capsule and incision before suturing the articular capsule. Then, the incision was closed layer by layer after placing a plasma drainage tube in the anterior iliac region.

The control group received combined spinal-epidural anesthesia combined with infiltration anesthesia. The approaches of infiltration anesthesia were the same as those in the experimental group, and the steps of conducting combined spinal-epidural anesthesia were as follows. Half an hour before the surgery, the patients were given 10 mg of diazepam (Biozen Pharmaceutical Co., Ltd., NMPA approval no. H41023114) and 0.5 mg of atropine (Grandpharma (China) Co., Ltd., NMPA approval no. H42021922). The operating table was adjusted to a head-high and foot-low position, and the two-point puncture method was adopted to treat the patients with epidural puncture tubes and punctures in spinal subarachnoid space. Then, 2.0% lidocaine was injected at the puncture points of L_1_-L_2_ or T_12_-L_1_, and the mixed liquor of 1 ml of 100% glucose solution and 2 ml of 0.5% bupivacaine (Zizhu Pharmaceutical Co., Ltd., NMPA approval no. H11020426) were injected at the L_2_-L_3_ puncture points. After the anesthesia, the patients took the horizontal position and the anesthetic plane was adjusted accordingly. If the spinal anesthesia could not meet the requirement for the surgical anesthetic plane, the patients would receive epidural administration.

All the patients were fasted routinely before surgery and given routine monitoring and a nasal catheter to inhale oxygen when entering the operation room. When the blood pressure of the patients in the two groups elevated or decreased by more than 30.0% of the base value, they were intravenously injected with 0.5-2.0 *μ*g/kg of nitroglycerin (Chuangchun Yishenkang Biopharmaceutical Co., Ltd., NMPA approval no. H22021894) and 1-2 *μ*g/kg of dopamine (Second Pharma Co., Ltd., NMPA approval no. H11020137), respectively.

### 2.5. Observational Indexes

1. The general data included patients' sex, age, body weight, body mass index (BMI), ASA grade, complications (hypertension, diabetes, chronic obstructive pulmonary disease, cerebral infarction and others), surgical approach (internal fixation surgery and noninternal fixation surgery), monthly income, education level, payment manner of medical expenses, and place of residence
2. The anesthesia comfort score was 0-10 points, with 0 as feeling comfortable and 10 as feeling the most uncomfortable. The patients' mood change (pain expression), shivering response, and traction reaction were observed, and the patients' anesthesia comfort was scored according to the scale of marks
3. Stress responses: venous blood (5 ml) was collected from each patient before anesthesia (*T*_1_), and at the end of surgery (*T*_2_), and 12 hours after surgery (*T*_3_). The blood glucose meter (Bayer Healthcare Company Limited, NMPA Certified No. 20092402599) was adopted to determine the patients' blood glucose levels. The enzyme-linked immunosorbent assay was adopted to determine the patients' cortisol levels and catecholamine levels, and the high-performance liquid chromatography electrochemical method was adopted to determine their norepinephrine levels. All the kits were bought from Beijing Kewei Clinical Diagnostic Reagent Inc., and the operations were conducted in strict accordance with the instructions
4. Postoperative pain indexes: the numerical rating scale (NRS) [13] was adopted to evaluate the patients' pain indexes at 12 (*T*_3_) and 24 hours after surgery (*T*_4_). This scale used the numbers 1-10 to present pain degrees. Namely, a straight line was equally divided into 10 segments, and 1-10 points were plotted from low to high, with 0 as no pain and 10 as severe pain that makes the patients unable to fall asleep

### 2.6. Statistical Treatment

The statistical software SPSS 20.0 was adopted for data processing and GraphPad Prism 7 (GraphPad Software, San Diego, USA) was used to draw graphs of the data in this study. This study included count data and measurement data, which were tested by *χ*^2^ and *t*. When *P* < 0.05, the differences were considered statistically significant.

## 3. Results

### 3.1. Comparison of the General Data

No statistical difference in patients' general data was found between the two groups (*P* > 0.05; Table 1).

### 3.2. Comparison of the Anesthesia Comfort Scores

**Figure 1:**
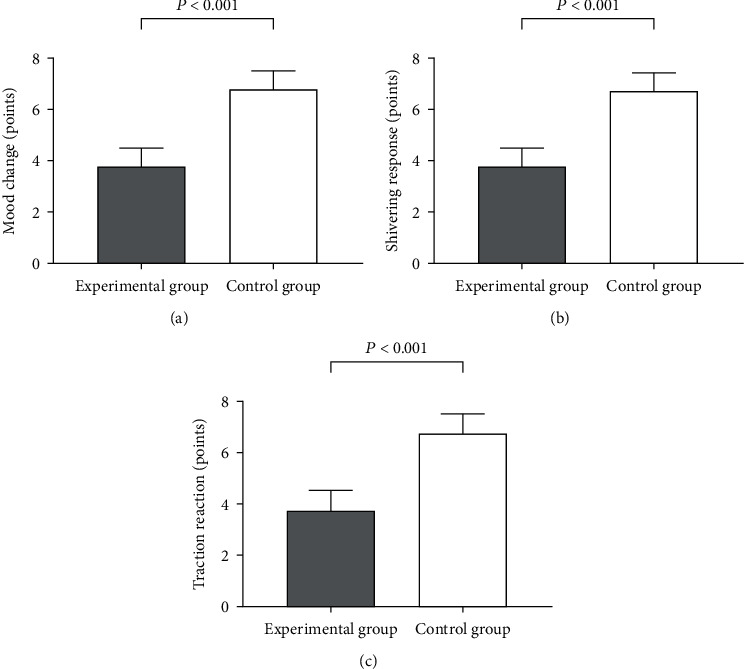
Comparison of the anesthesia comfort scores (*x* ± *s*, points). Notes: (a) the comparison of mood changes, (b) the comparison of shivering responses, and (c) the comparison of traction reactions.

Compared with the control group, the experimental group achieved much lower scores of mood change, shivering response, and traction reaction (*P* < 0.001), indicating that the anesthesia comfort in the experimental group was higher (Figure 1).

The experimental group achieved much lower scores of mood change, shivering response, and traction reaction compared with the control group (3.80 ± 0.72 vs. 6.76 ± 0.74, 3.76 ± 0.74 vs. 6.68 ± 0.68, and 3.74 ± 0.80 vs. 6.74 ± 0.77; *P* < 0.001).

### 3.3. Comparison of the Stress Responses

**Figure 2:**
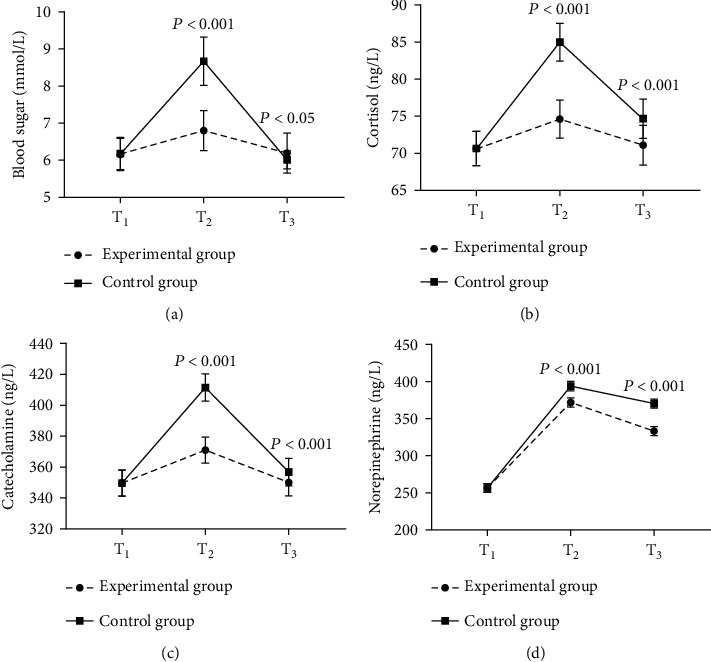
Comparison of the stress responses (*x* ± *s*). Notes: (a) the comparison of blood sugar levels (mmol/l), (b) the comparison of cortisol levels (ng/l), (c) the comparison of catecholamine levels (ng/l), and (d) the comparison of norepinephrine levels (ng/l).

Compared with the control group, the experimental group had much better perioperative stress response indexes (*P* < 0.05; Figure 2).

No remarkable difference in blood sugar levels at *T*_1_ between the experimental group and the control group was found (6.15 ± 0.45 vs. 6.18 ± 0.44, *P* = 0.737). The experimental group achieved much lower blood sugar level at *T*_2_ compared with the control group (6.80 ± 0.54 vs. 8.67 ± 0.65, *P* < 0.001). The experimental group achieved much higher blood sugar level at *T*_3_ compared with the control group (6.19 ± 0.54 vs. 6.01 ± 0.24, *P* = 0.034).

No remarkable difference in cortisol levels at *T*_1_ between the experimental group and the control group was found (70.65 ± 2.14 vs. 70.67 ± 2.35, *P* = 0.965). The experimental group achieved much lower cortisol levels at *T*_2_ and *T*_3_ compared with the control group (74.65 ± 2.47 vs. 84.98 ± 2.58, 71.11 ± 2.65 vs. 74.68 ± 2.57; *P* < 0.001).

No remarkable difference in catecholamine levels at *T*_1_ between the experimental group and the control group was found (349.65 ± 8.41 vs. 349.44 ± 8.65, *P* = 0.902). The experimental group achieved much lower catecholamine levels at *T*_2_ and *T*_3_ compared with the control group (370.65 ± 8.41 vs. 410.98 ± 8.74, 349.65 ± 8.74 vs. 355.94 ± 8.41; *P* < 0.001).

No remarkable difference in norepinephrine levels at *T*_1_ between the experimental group and the control group was found (254.75 ± 4.68 vs. 254.80 ± 4.69, *P* = 0.958). The experimental group achieved much lower norepinephrine levels at *T*_2_ and *T*_3_ compared with the control group (370.65 ± 5.65 vs. 392.65 ± 5.74, 332.21 ± 5.98 vs. 369.65 ± 5.741; *P* < 0.001).

### 3.4. Comparison of Postoperative Pain Indexes

The experimental group had much lower pain scores at 12 (*T*_3_) and 24 (*T*_4_) hours after surgery compared with the control group (2.08 ± 0.72 vs. 2.50 ± 0.75, 1.22 ± 0.41 vs. 1.62 ± 0.69; *P* = 0.005 and 0.001).

## 4. Discussion

The World Health Organization lists pain as the fifth vital sign of the human body [14]. Intense pain seriously affects the patients' physical and mental health by inhibiting the normal function of many systems, like respiratory and cardiovascular systems. Clinical practice has shown that patients undergoing hip replacement feel moderate and severe pain after surgery [15]. Especially at 24 hours after the surgery, the patients feel more obvious pain. Besides, the surgery and anesthesia make them feel fearful, so they are loath to cooperate with the functional rehabilitation training, which is not conducive to their early recovery after surgery [16]. The compensatory capacity and organ function of elderly patients undergoing hip replacement have declined, and the noxious stimulation from anesthesia and surgery on such capacity and function is more obvious. Besides, the intense stress response further increases the patients' physical and mental burden [17]. A stress response is a general adaptation syndrome that occurs when the organism is exposed to noxious stimulation. A moderate stress response is beneficial to maintain the stability of the intraoperative vital signs, while the excessive stress response can lead to the dysfunction of neurological, endocrine, and immune systems and cause serious complications. Therefore, reducing the stress responses of elderly patients undergoing hip replacement has important implications in relieving their postoperative pain and speeding up their recovery.

Anesthesia is a key element that affects the stress response, and different types of anesthesia and anesthetic drugs have different effects on the stress response [18, 19]. Multimodal balanced analgesia is a relatively advanced analgesic method, aimed at exerting comprehensive analgesic effects by adopting multiple types of anesthesia and anesthetic drugs. Multimodal balanced analgesia has been applied in hip replacement, but different routes of administration and drugs are used in patients, so the effects on reducing stress response are various [20, 21]. The lumbar plexus block selected in this study is a commonly used anesthetic method in hip replacement, and it is more conducive to reducing hemodynamic fluctuations and effectively maintaining the stability of elderly patients' signs compared with general anesthesia and intraspinal block. With the development of ultrasound technology in recent years, physicians have a better grasp of the structure of the target nerves and the routes of the puncture needle, so success rates of puncture and injection have increased substantially [22]. It is worth noting that hip surgery affects the areas beyond the lumbar plexus, so the lumbar plexus block alone is likely to cause discomfort and needs to be combined with sedation. However, elderly patients are likely to develop respiratory depression with excessive sedation [23], so the lumbar plexus block should be applied in combination with other more effective analgesia methods. This study combined dexmedetomidine with local infiltration anesthesia, which anesthetizes the injection site by local injection and is conducive to reducing postoperative incision pain. According to the study of scholars Brendan et al., the application of the multimodal analgesia regimen which takes the local infiltration anesthesia as the core in total hip replacement effectively reduces the dosage of opioids after surgery [24] and accelerates the patients' recovery.

According to this study, the local infiltration anesthesia with lumbar plexus block was more effective than the combination of combined spinal-epidural anesthesia and infiltration anesthesia, and the patients in the experimental group had better anesthesia comfort. The reasons for the above study result are as follows. For elderly patients, their arachnoid villi are increased and their nerve roots are compressed, so the connective tissue proliferation leads to foraminal stenosis. As a result, it is difficult to control the upper bound of block levels of spinal anesthesia, and it is likely to have too high block levels. At the same time, the patients' blood vessels are dilated after anesthesia, and their hemodynamic changes are more obvious, which affects their respiratory and circulatory functions. Therefore, the patients receiving combined spinal-epidural anesthesia have lower comfort compared with the patients receiving lumbar plexus block. The single lumbar plexus block can block the affected limb and has less impact on the circulation, so the patient has fewer hemodynamic fluctuations and more stable signs. The catecholamine level is an important indicator of the body's hemodynamics and stress level. The experimental group had lighter stress responses, so the catecholamine levels in this group were lower. According to scholars Choi et al., elderly patients undergoing lower abdominal surgery who received lumbar plexus block had lower catecholamine levels compared with those who received general anesthesia, indicating that lumbar plexus block has positive effects on maintaining the signs of elderly patients [25]. In addition to catecholamine, blood glucose, cortisol, and norepinephrine also reflect the stress responses. In order to reduce the effect of time on cortin, this study scheduled the surgery for ten o'clock in the morning. It was found that the experimental group achieved remarkably better stress response indexes, indicating that the lumbar plexus block has a definite effect on reducing stress responses and can be applied in elderly patients undergoing hip replacement. This study also has some limitations. Firstly, it did not explore the incidence of adverse reactions in both groups. But in the related studies of elderly patients, anesthesia safety is a topic worthy of discussion. Secondly, this study has a small sample size possibly due to the restrains of patients' actual situation and geographical factors. Thirdly, since the study did not consider geographical factors, the research results may only reflect the situation of hip replacement in the region. Therefore, more subsequent studies should be conducted with an expanded sample size for deeper exploration.

In conclusion, lumbar plexus block combined with infiltration anesthesia can relieve the stress responses and postoperative pain of elderly patients undergoing hip replacement and increase their anesthesia comfort. Therefore, this anesthesia method is translational in clinic.

## Figures and Tables

**Table 1:** Comparison of the general data.

Groups	Experimental group (*n* = 50)	Control group (*n* = 50)	*χ* ^2^/*t*	*P*
Male/female	30/20	28/22	0.164	0.685
Age (years old)	70.32 ± 5.28	70.76 ± 5.32	0.415	0.679
Body weight (kg)	64.65 ± 2.55	65.01 ± 2.47	0.717	0.475
BMI (kg/m^2^)	22.68 ± 2.41	22.74 ± 2.68	0.118	0.907
ASA grades				
I	12	10	0.233	0.629
II	28	27	0.040	0.841
III	10	13	0.508	0.476
Complications				
Hypertension	20	18	0.170	0.680
Diabetes	15	16	0.047	0.829
Chronic obstructive pulmonary disease	8	10	0.271	0.603
Cerebral infarction	5	4	0.122	0.727
Others	8	10	0.271	0.603
Surgical approach			0.040	0.841
Internal fixation surgery	25	26		
Noninternal fixation surgery	25	24		
Month income (yuan)			0.041	0.839
≥4000	21	20		
<4000	29	30		
Education level			0.407	0.523
Senior high school and below	35	32		
College and higher	15	18		
Payment manner of medical expenses				
Medical insurance	18	20	0.170	0.680
Commercial insurance	20	22	0.164	0.685
Self-pay and others	12	8	1.000	0.317
Place of residence			0.040	0.841
Urban area	28	27		
Rural areas	22	23		

## Data Availability

Data to support the findings of this study is available on reasonable request from the corresponding author.
